# Changes in Responses of Neurons in Spinal and Medullary Subnucleus Reticularis Dorsalis to Acupoint Stimulation in Rats with Visceral Hyperalgesia

**DOI:** 10.1155/2014/768634

**Published:** 2014-11-26

**Authors:** Ling-Ling Yu, Liang Li, Pei-Jing Rong, Bing Zhu, Qing-Guang Qin, Hui Ben, Guo-Fu Huang

**Affiliations:** ^1^Department of Acupuncture and Moxibustion, Wuhan Hospital of Integrated Chinese and Western Medicine, 215 Zhongshan Road, Wuhan 430022, China; ^2^Institute of Acupuncture and Moxibustion, China Academy of Chinese Medical Sciences, Beijing 100700, China

## Abstract

The purpose of this study was to explore the mechanism of acupoints sensitization phenomenon at the spinal and medulla levels. Experiments were performed on adult male Sprague-Dawley rats and visceral noxious stimuli was generated by colorectal distension (CRD). The activities of wide dynamic range (WDR) and subnucleus reticularis dorsalis (SRD) neurons were recorded. The changes of the reactions of WDR and SRD neurons to electroacupuncture (EA) on acupoints of “Zusanli-Shangjuxu” before and after CRD stimulation were observed. The results showed that visceral nociception could facilitate the response of neurons to acupoints stimulation. In spinal dorsal horn, EA-induced activation of WDR neurons further increased to 106.84 ± 17.33% (1.5 mA) (*P* < 0.001) and 42.27 ± 13.10% (6 mA) (*P* < 0.01) compared to the neuronal responses before CRD. In medulla oblongata, EA-induced activation of SRD neurons further increased to 63.28 ± 15.96% (1.5 mA) (*P* < 0.001) and 25.02 ± 7.47% (6 mA) (*P* < 0.01) compared to that before CRD. Taken together, these data suggest that the viscerosomatic convergence-facilitation effect of WDR and SRD neurons may underlie the mechanism of acupoints sensitization. But the sensitizing effect of visceral nociception on WDR neurons is stronger than that on SRD neurons.

## 1. Introduction

Due to different structural function units of related somites, there exist specific relationships between acupoints and target organs. While previous studies on the relationship of acupoints and organs mostly focus on the acupoints' regulation of visceral function in healthy state neglecting its function of reflecting visceral diseases, recently, Yu et al. [1] put forward the concept of “dynamic states of acupoints,” deeming that the size and function of acupoints are not static but change dynamically. The function and size of acupoints will vary along with the state of the body. Clinical studies have shown that the pressure-pain threshold of related acupoints decreases in patients with functional intestinal disorder [2] and gastric ulcer [3]. These findings suggest that acupoint is a dynamic functional unit and can be sensitized when viscera is under pathological process.

Many features of the acupoint sensitization phenomenon resemble the features of referred pain in Western medicine. Referred pain is an important symptom of visceral irritation or inflammation [4–6]. It is characterized by visceral hypersensitivity, expanded dermatomes of referred sensation, and somatic hyperalgesia including the presence of tenderness on examination of the skin or muscle [7, 8]. Many studies have shown that referred pain often occurs in body regions somatotopically different from the sites of target organs [9, 10] and has segmental pattern related to target organs [11], suggesting the involvement of central hyperalgesic mechanisms [12]. Our working hypothesis is that central sensitization produce the increased pain sensitivity in sensitized acupoints.

Both the wide dynamic range (WDR) neurons [13, 14] in spinal dorsal horn and the subnucleus reticularis dorsalis (SRD) neurons [15] in the caudal portion of the medulla could be activated by afferent signals from acupoints and inner organs. Many morphological studies observed the neuronal projections between spinal cord and SRD area [16–20], suggesting the existence of a neuronal circuit interconnecting the spinal cord and the medulla oblongata. In our previous studies [21, 22], we observed that visceral inflammation could facilitate the responses of WDR and SRD neurons to acupoint stimulation and a linear relationship existed between the intensity of nociceptive stimulation to inner organs and the sensitivity of related acupoint. These findings suggest that the function of viscerosomatic convergence-facilitation at the spinal and medulla levels maybe related to acupoint sensitization phenomenon.

To further test the different role of WDR and SRD neurons in the dynamic change of the sensitivity of acupoints, in this study, we compared the responses of WDR and SRD neurons to acupoint stimulation in rats before and after receiving visceral nociception. The aim of this study was to obtain mechanism of acupoint sensitization phenomenon at spinal cord and medulla levels.

## 2. Method

### 2.1. Animals and Preparation

The experiments were performed on adult male Sprague-Dawley rats (body weight 250–280 g), obtained from the Laboratory Animal Center of China Academy of Military Medical Sciences (License number: SCXK- (Military) 2007–004). Rats were housed in standard laboratory conditions under artificial 12 h light/dark cycle and an ambient temperature of 22 ± 0.5°C for one week. Food and water were available ad libitum. Following an intraperitoneal injection of 100 *μ*g atropine sulfate, the animals were anesthetized with an intraperitoneal injection of 10% urethane (1.0–1.2 g/kg, ip) and artificially ventilated through a tracheal cannula. The body temperature of animals was maintained at 37 ± 0.5°C by means of a feedback-controlled homeothermic heating blanket system (RWD CL-8).

All experimental procedures were approved in accordance with the* Guide for the Care and Use of Laboratory Animals* issued by China Academy of Chinese Medical Science.

### 2.2. Animal Surgeries

#### 2.2.1. Surgery on Spinal Cord

A laminectomy was performed at spinal segments L1–L3 and the corresponding vertebrae mounted on a rigid frame. The spinal cord was exposed by removing the spinal dura mater and pia mater.

#### 2.2.2. Surgery on Medulla Oblongata

The animals were mounted in a stereotaxic frame with the head fixed in a ventroflexed position by a metallic bar cemented to the skull. The caudal medulla was exposed by removing the overlying musculature and dura mater.

After the surgeries, the flap was sewn into skin cell and covered with 38°C paraffin oil to avoid drying.

### 2.3. Experimental Procedure

#### 2.3.1. Recordings

Unitary extracellular recordings were made with glass micropipettes filled with a mixture of 2% pontamine sky blue and 0.1 M of natrium aceticum (cusp: 5 *μ*m, impedance: 8–12 MΩ). According to the three-dimensional spatial coordinates of the nucleus, the micropipettes were inserted into the nuclei under the control of micropipette manipulator (SM-21, Japan). The coordinate of WDR neuron is 0.5–1.5 mm lateral to the midline of the back of spinal cord and 500–1500 *μ*m beneath the surface of spinal cord. The coordinate of SRD neuron is 1.0–2.0 mm caudal to the obex and 0.5–1.5 mm lateral to the midline of medulla oblongata.

Single-unit activities were fed into a window discriminator and displayed on an oscilloscope screen. Outputs of the window discriminator and amplifier were led into a data collection system (PowerLab) and a personal computer-based data acquisition system (Chart 5.0) to compile histograms or wavemark files.

#### 2.3.2. Identification of Neurons

Nonnoxious and noxious electrical stimuli were used to isolate unitary activities, and the skin receptive fields were mapped by means of gentle tapping and brushing stimuli. The recorded neurons were classified on the basis of their responses to different stimuli applied on their peripheral receptive fields. At the experiment on spinal cord, neurons that could be activated by both nonnoxious and noxious stimuli applied to the skin receptive fields were identified as WDR neurons. At the experiment on medulla oblongata, neurons that could be activated by noxious stimuli applied to any part of the skin but could not be activated by nonnoxious stimuli were identified as SRD neurons.

#### 2.3.3. Stimulation

Visceral nociceptive stimulation was generated by colorectal distension. A 4–6 cm long inflatable balloon catheter was inserted into rats' colorectum with a depth of 4 cm. CRD stimulation was carried out by pressure supplied by an 80 mmHg sphygmomanometer for 60 s. In order to prevent possible sensitization triggered by overstimulation to the colorectum, the interval between two CRD stimulations was at least 10 minutes. Other technical details have been described in our previous reports.

EA stimulation was applied at the acupoints of “Zusanli-Shangjuxu” at the recorded homolateral discharging neurons with the frequency of 15 Hz for 30 seconds. EA stimulation output comes from stimulator (88–102 G, Nihon Kohden) and the intensity was 1.5 mA and 6 mA.

#### 2.3.4. Recordings Procedures

A standard conditioned recording procedure was set as follows: the background activity was recorded for 60 s, and from 30 s to 60 s the EA stimuli were administered. After 60 s of recovery time for neuronal discharge, neuron activity was recorded for 30 s followed by a test of the neurons' responses to CRD. After another 60 s of recovery of neuronal discharge, EA stimuli with the same intensity were applied at the acupoint before CRD, and the responses of neurons to EA after CRD stimuli were observed.

### 2.4. Histological Location

After single-unit recording, the recording sites were marked by electrophoretic deposition of pontamine sky blue and checked by HE coloration. Locations of the recording sites were then determined with reference to the brain atlas of the rat (Paxinos & Watson, 2007).

### 2.5. Data Collection and Statistical Analysis

Neuronal discharges per second and the activation/suppression ratio (identified as $\overset{-}{X}\pm \text{SE}$%) were calculated with PowerLab, Chart 5.0, and SPSS13.0. Descriptive analyses were carried out for the average and differences of the pre- and postintervention data (identified as $\overset{-}{X}\pm \text{SE}$%). Paired *t*-test was used for cross-group comparison. *P* < 0.05 is deemed statistically significant.

## 3. Results

### 3.1. Results of Experiment on Spinal Cord

#### 3.1.1. General Features of WDR Neurons

**Figure 1:**
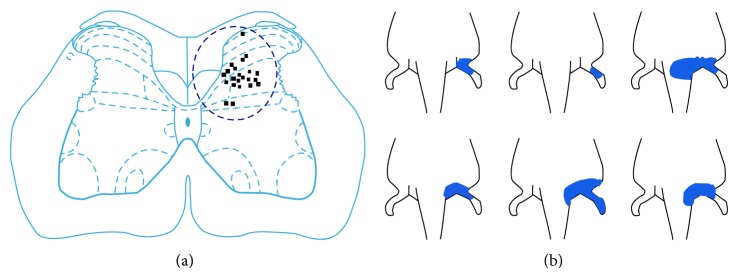
(a) Positions of WDR neurons marked by pontamine sky blue in the spinal dorsal horn. (b) The skin receptive fields of WDR neurons.

30 WDR neurons were well isolated from the background. Most of the WDR neurons were located in laminas IV and V, and a few in laminas I and VI of the gray matter marked by electrophoresis of the pontamine sky blue dye at the end of experiment (see Figure 1(a)). The skin receptive fields of the majority of the WDR neurons responding to somatic stimuli were distributed along the ipsilateral caudal parts of the body, including the scrotum, hip region, tail root, hind limb, and the hind paw (see Figure 1(b)).

**Figure 2:**
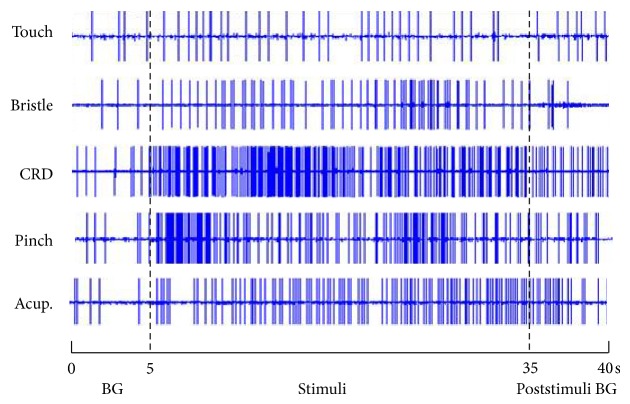
The response patterns of WDR neurons to different stimuli applied at skin receptive fields and viscera. The whole recording time was 40 s, of which the first 5 s was the duration for recording spontaneous background (BG) discharges; from the 5th to 35th s, the stimuli of touch, bristle, 80 mmHg CRD, pinch, and acupuncture were consecutively administered; the poststimuli BG discharges were recorded from the 35th to 40th s.

When a neuron recorded, we observed it's response to different stimuli applied at skin receptive fields and viscera.  Figure 2 illustrated that WDR neurons not only responded to nonnoxious stimulation (such as stimuli of touch and bristle), but also responded significantly to noxious stimulation (such as stimuli of pinch and acupuncture) applied on skin receptive fields. We recorded 8 WDR neurons and observed their discharge reactions induced by 80 mmHg CRD stimulation. After CRD, the neuronal discharges increased from 2.48 ± 0.57 spikes/s of the background to 6.71 ± 1.80 spikes/s. The increasing rate was 171.97 ± 47.55% (*P* < 0.001), indicating that 80 mmHg CRD stimulation could activate the activity of WDR neurons. WDR neurons that could be activated by both EA at acupoints of “Zusanli-Shangjuxu” and CRD were chosen as research neurons for further experiment.

#### 3.1.2. Comparison of the Effects of EA on WDR Neurons before and after CRD

**Figure 3:**
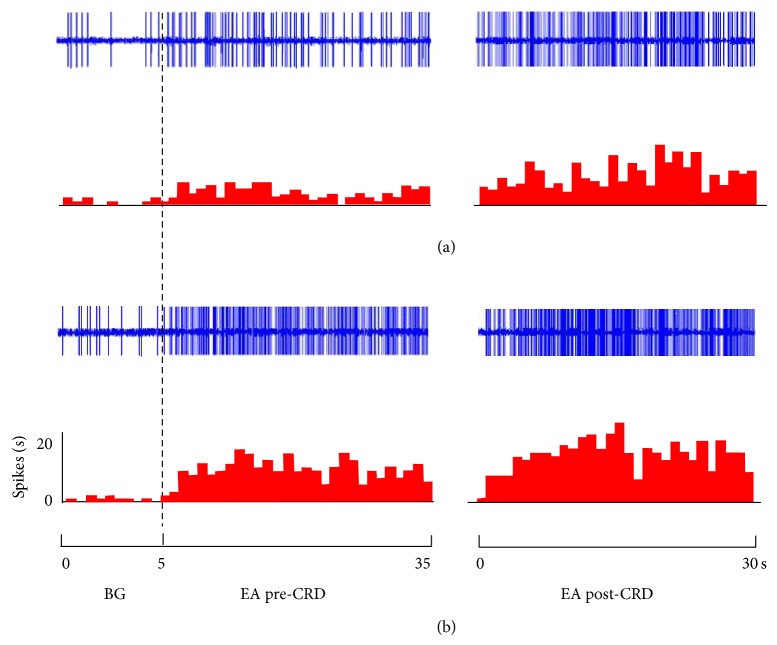
Responses of WDR neurons to EA at different intensities ((a) 1.5 mA, (b) 6 mA) before and after CRD. Upper rows show original unit discharges and lower rows show histograms. BG shows spontaneous activities. EA pre-CRD shows neuronal activities induced by EA before CRD. EA post-CRD shows neuronal activities induced by EA after CRD stimuli.

Different intensities of EA were applied at acupoints of “Zusanli-Shangjuxu” before and after rats were given 80 mmHg CRD for 60 s. The responses of WDR neurons to EA before and after CRD were observed. Results showed that the numbers of discharges of WDR neurons evoked by EA were significantly increased after CRD (see Table 1 and Figure 3).

**Figure 4:**
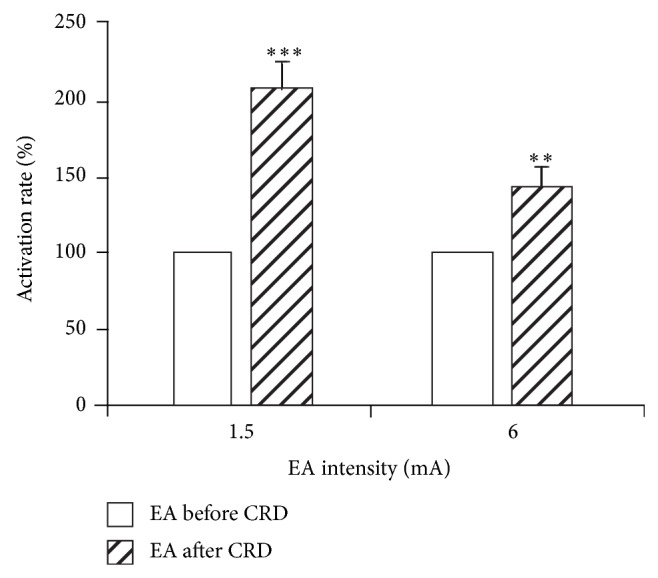
The activation rate of WDR neurons induced by EA at different intensities before and after CRD (left: before; right: after). After CRD, EA stimulation further increased the discharges of WDR neurons to EA (^**^ *P* < 0.01, ^***^ *P* < 0.001).

When EA intensity was set at 1.5 mA, before CRD, the activation rate of WDR neurons caused by EA was 39.42 ± 11.10%, while after CRD, the activation rate of EA with the same intensity rose to 188.44 ± 33.54%, with a further increasing rate of 106.84 ± 17.33% compared to its effect before CRD. There was a very significant difference before and after CRD (*P* < 0.001) (see Figure 4).

When EA intensity was set at 6 mA, the activation rates of WDR neurons caused by EA were 206.67 ± 38.30% and 333.79 ± 46.40% before and after CRD, respectively, with a further increasing rate of 42.27 ± 13.10% after CRD. There was a significant difference before and after CRD (*P* < 0.01) (see Figure 4).

### 3.2. Results of Experiment on Medulla Oblongata

#### 3.2.1. General Features of SRD Neurons

**Figure 5:**
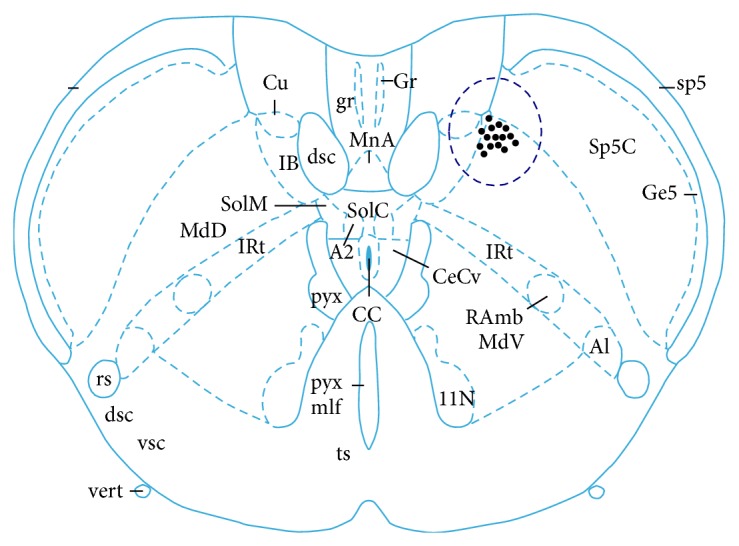
Positions of SRD neurons marked by pontamine sky blue in the dorsal medulla.

**Figure 6:**
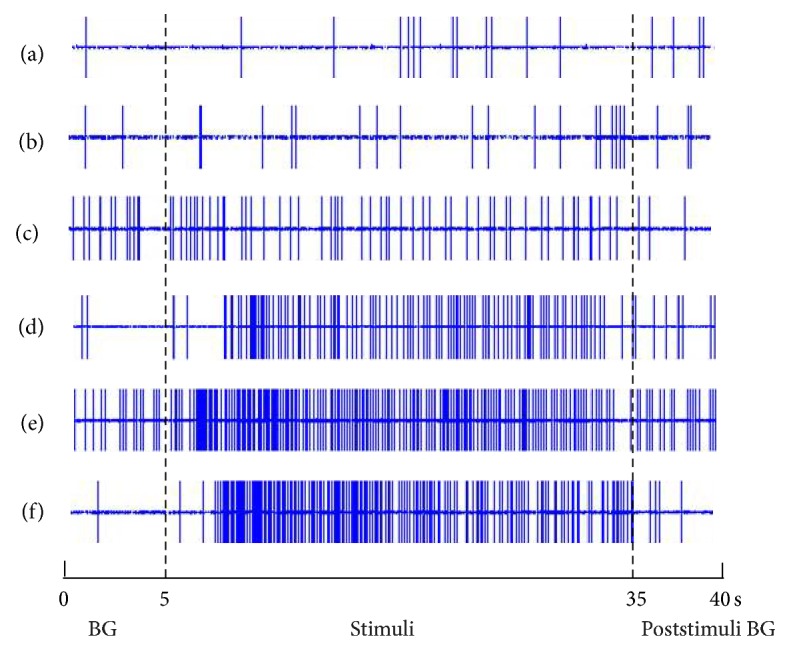
The responses of SRD neurons to different stimuli from skin receptive fields and viscera. The whole recording time was 40 s, of which the first 5 s was the duration for recording spontaneous background (BG) discharges; from the 5th to 35th s, the stimuli of (a) touch, (b) the bristles stimuli, (c) 1.5 mA EA, (d) pinch, (e) CRD, and (f) 4 mA EA were consecutively administered; the poststimulus BG discharges were recorded from the 35th to the 40th s.

28 SRD neurons were recorded in the dorsal medulla and their positions were mapped by electrophoretic deposition of pontamine sky blue at the end of experiments (see Figure 5). The responses of neurons to touch, brush bristles, pinch, CRD, and different intensities of EA stimulation were observed. Results showed that SRD neurons had significant responses to noxious stimulations (such as pinch, CRD, and 4 mA EA) but had no responses to any kind of nonnoxious stimulations (such as touch, brush bristles, and 1.5 mA EA) (see Figure 6).

#### 3.2.2. Comparison of the Effects of EA on SRD Neurons before and after CRD

We recorded 8 SRD neurons and observed their discharge reactions induced by 80 mmHg CRD stimulation. After CRD, the neuronal discharges increased from 3.10 ± 0.69 spikes/s of the background activity to 13.73 ± 2.24 spikes/s. The increasing rate was 352.32 ± 68.03% (*P* < 0.001). It indicated that nociceptive CRD stimulation could significantly activate the activity of SRD neurons.

**Figure 7:**
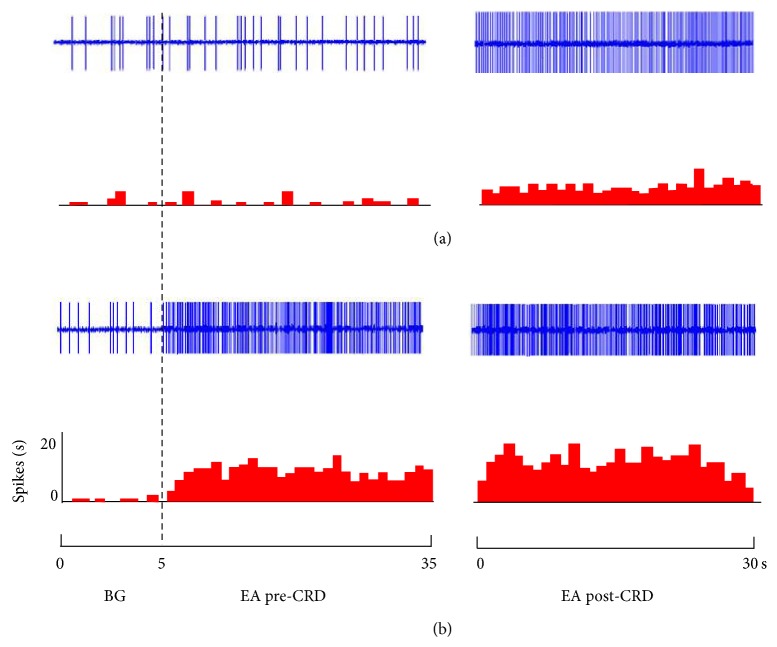
The response patterns of SRD neurons to EA before and after CRD. (a) A neuron not activated by 1.5 mA before CRD but activated after CRD. (b) A neuron activated by 6 mA both before and after CRD but showing a stronger response to EA after CRD.

Different intensities of EA were applied at acupoints of “Zusanli-Shangjuxu” before and after rats were given 80 mmHg CRD for 60 s.  Figure 7 indicates that the responses of SRD neurons to EA before and after CRD were different. After CRD, the numbers of discharges of SRD neurons evoked by EA of the same intensity before CRD were also significantly increased (see Table 2).

**Figure 8:**
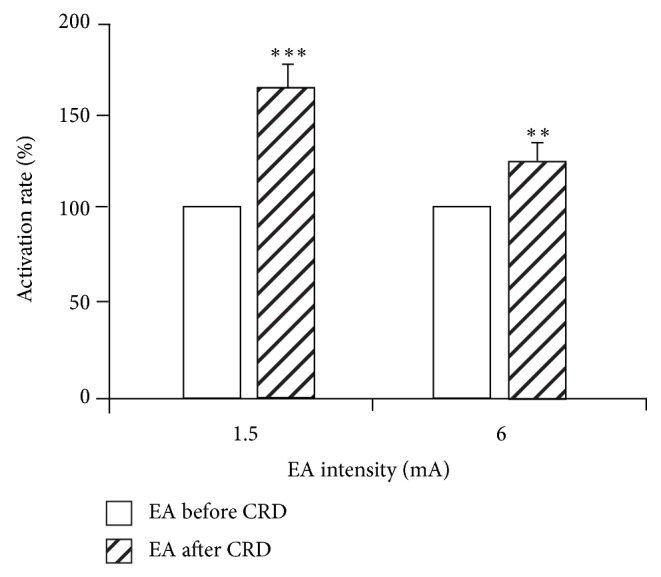
The activation rate of SRD neurons induced by EA at different intensities before and after CRD (left: before; right: after). After CRD, EA stimulation further increased the discharges of SRD neurons (^**^ *P* < 0.01, ^***^ *P* < 0.001).

Responses of 10 SRD neurons to nonnoxious EA stimulation (1.5 mA) before and after CRD were compared. Before CRD, SRD neurons had no activating reaction to EA compared with background activity (*P* > 0.05), but after CRD, EA had significant activating effect on SRD neurons. Compared to the background activity, the activating rate was 67.59 ± 21.68% (*P* < 0.001). Compared to the effect of EA before CRD, the activating rate was 63.28 ± 15.96%; there was a very significant difference in the effect of EA before and after CRD (*P* < 0.001) (Figure 8).

Responses of anther 11 SRD neurons to noxious EA stimulation (6 mA) before and after CRD were compared. Results showed that 6 mA EA stimulation had significant activating effect on SRD neurons both before and after CRD, but a stronger activation effect was observed after CRD. The neuronal discharge increased to 25.02 ± 7.47% after CRD compared with that before CRD. There was a significant difference in the effect of EA before and after CRD (*P* < 0.01) (Figure 8).

## 4. Discussion

Acupoints are regarded as specific spots on the body surface where Qi from meridian and viscera is infused. It is believed that acupoints have dual functions of diagnosis and treatment of visceral diseases. Pathological changes in viscera can be manifested on the body surface and cause the sensitization of acupoints with various pathological reactions mainly led by pain when pressed. The size and function of acupoints will vary along with the state of viscera. The so-called acupoint sensitization phenomenon is the specific reflection of visceral diseases through acupoints [21, 22].

Recently, some researchers have focused on the phenomenon of acupoint sensitization. Morphological studies showed that, under inflammatory state, sick organs can promote the extravasation of Evans blue on the body surface. For rats with ovarian inflammation, extravasated EB points mainly distribute around the “Guanyuan (RN4)”-“Uterus” area and the “Shenshu (BL23)”-“Mingmen (DU4)” area [23]. For rats with acute gastric mucosa inflammation, extravasated EB points distribute along with nerve segments and highly coincide with “Pishu (BL20),” “Shenshu (BL23),” and nearby acupoints [24]. But the extravasation of EB points is rarely observed on healthy rats. It indicates that acupoint is a dynamic functional unit. When the organs change from the healthy state to the pathological state, acupoints shift from the silent model to the sensitized model.

The mechanism of acupoint sensitization phenomenon is largely unknown. But both acupoints' functions of diagnosis and treatment of visceral diseases are related to the convergence of visceral and somatic afferent at spinal cord and/or supraspinal centrum. Results of the present study showed that nociceptive CRD stimulation could facilitate the response of WDR and SRD neurons to EA stimulation applied to acupoints “Zusanli-Shangjuxu.” It is in accordance with the convergence-facilitation mechanism that explains referred pains. Many studies have shown that the body-viscera convergent neurons in the spinal dorsal horn and supraspinal centrum can be sensitized by stimulation from the inner organs, and the sensitized convergent neurons' responses to inputs from the body surface also become stronger. For instance, for animals with referred muscle hyperalgesia caused by ureteral calculus, the number and frequency of background discharges of spinal cord cells exceed those of normal animals [25]; after chemical stimulation in the bladder, the background discharge level of neurons in the dorsal horn of the spinal cord is elevated [26]; compared with normal rats, rats with ureteral calculus have larger amount and higher frequency of background discharges in neurons from the dorsal horn of the spinal cord; the authors considered it as referred hyperalgesia [27]. Besides, inflammation in the esophagus [28] and colon [29] may lead to a reduction in response threshold. These findings suggest that the function of viscerosomatic convergence-facilitation at spinal cord (WDR) and medulla oblongata (SRD) may underlie the mechanism of acupoint sensitization.

In addition, large numbers of morphological studies [16–20] observed the neuronal projection between the spinal cord and the SRD area. Experiment compared the responses of WDR and SRD neurons to acupoint stimulation in rats before and after receiving visceral nociception. Results showed that spinal cord (WDR) and medulla oblongata (SRD) play different roles in acupoint sensitization. The sensitizing effect of visceral nociception on WDR neurons is stronger than that on SRD neurons. Therefore, we concluded that the neuronal projections between the spinal cord and the SRD area may form a neuronal circuit and play an important role in the interaction of somatic inputs and visceral inputs. These studies provided scientific lines of evidence for the phenomenon of referred pain in Western medicine and acupoint sensitization in traditional Chinese medicine.

The original meaning of acupoints is the locations that “cause pain or ease when pressed.” The original definition completely covers the two basic functions of acupoints: diagnosis (cause of pain) and treatment (ease). When visceral organs change from the physiological state to the pathological state, the corresponding acupoints on the body surface change from the silent model to the sensitized model. The phenomenon is the concrete manifestation of acupoints' function of diagnosis and treatment of diseases. We hypothesized that activated acupoints are not only the new points of pathological reaction on body surface, but also the best points for the treatment of visceral diseases. The analgesia effect of activated acupoints for corresponding internal organs is better than silent acupoints. But further studies are needed to test the hypothesis.

## 5. Conclusion

The above results indicated that visceral nociception could facilitate the response of WDR and SRD neurons to acupoints stimulation. The function of viscerosomatic convergence-facilitation at spinal cord and medulla oblongata may underlie the mechanism of acupoint sensitization. But spinal cord and medulla oblongata play different roles in acupoint sensitization. The sensitizing effect of visceral nociception on WDR neurons is stronger than that on SRD neurons.

## Figures and Tables

**Table 1:** The numbers of discharges of WDR neurons evoked by EA before and after CRD.

EA intensity (mA)	*n*	Background activity (spikes/s)	EA before CRD (spikes/s)	EA after CRD (spikes/s)
1.5	11	2.62 ± 0.50	3.64 ± 0.63	7.46 ± 0.92
6	11	2.74 ± 0.31	8.35 ± 0.98	11.80 ± 1.06

**Table 2:** The numbers of action potentials of SRD neurons evoked by EA before and after CRD.

Intensity	*N*	Background activity (spikes/sec)	EA before CRD (spikes/sec)	EA after CRD (spikes/sec)
1.5 mA	10	2.99 ± 0.47	3.09 ± 0.61	4.99 ± 0.87
6 mA	10	2.84 ± 0.42	11.15 ± 0.98	13.93 ± 1.45
